# Naoxintong/PPAR*α* Signaling Inhibits H9c2 Cell Apoptosis and Autophagy in Response to Oxidative Stress

**DOI:** 10.1155/2016/4370381

**Published:** 2016-09-07

**Authors:** Huimin Xu, Jianhua Jin, Lu Chen, Chunxiao Li, Qinggang Xu, Juanjuan Shi, Buchang Zhao, Yongzhong Hou, Hong Wang

**Affiliations:** ^1^Department of Oncology, Affiliated Wujin People's Hospital, Jiangsu University, Changzhou 212017, China; ^2^Institute of Life Sciences, Jiangsu University, Zhenjiang, Jiangsu 212013, China; ^3^Tianjin State Key Laboratory of Modern Chinese Medicine, Tianjin University of Traditional Chinese Medicine, 312 Anshanxi Road, Nankai District, Tianjin 300193, China; ^4^Key Laboratory of Pharmacology of Traditional Chinese Medical Formulae, Tianjin University of Traditional Chinese Medicine, Ministry of Education, Tianjin 300193, China; ^5^Xi'an Buchang Cardio-Cerebrovascular Disease Hospital, Xi'an 710003, China; ^6^Tianjin Key Laboratory of Traditional Chinese Medicine Pharmacology, Tianjin University of Traditional Chinese Medicine, 88 Yuquan Road, Nankai District, Tianjin 300193, China

## Abstract

Naoxintong (NXT) is an empirical formula based on the principle of traditional Chinese medicine, which has been approved by China Food and Drug Administration (CFDA) and is widely used for treatment of patients with cerebrovascular and cardiovascular diseases in China. The aim of this study is to investigate the protective mechanism of NXT on H9c2 cells (cardiogenic cell line) in response to H_2_O_2_. MTT, Western blot, and flow cytometry (FCM) methods were used to identify the protective effect of NXT extract on H_2_O_2_-induced H9c2 cells. Here we found that NXT extract significantly increased H9c2 cell viability and reduced H_2_O_2_-induced cell apoptosis and autophagy. More importantly, NXT inhibited H_2_O_2_-induced H9c2 cell apoptosis and autophagy by increasing PPAR*α* protein levels. In contrast, silenced PPAR*α* terminated NXT protective effect on H_2_O_2_-induced H9c2 cells. These findings suggest that NXT/PPAR*α* signaling suppressed H_2_O_2_-induced H9c2 cell apoptosis and autophagy.

## 1. Introduction

Naoxintong (NXT) is an empirical formula based on the principle of traditional Chinese medicine, which has been approved by China Food and Drug Administration (CFDA, Z20025001) and widely used for treatment of patients with cerebrovascular and cardiovascular diseases in China. NXT contains sixteen kinds of traditional Chinese medicines including* Astragalus membranaceus* (Fish.) Bge. (Huangqi),* Radix Paeoniae* (Chishao),* Salviae miltiorrhizae radix et rhizoma *(Danshen),* Radix Angelicae Sinensis* (Danggui),* Radix Paeoniae Rubra *(Chishao),* Rhizoma Ligustici Chuanxiong* (Chuanxiong),* Semen Persicae* (Taoren),* Achyranthes bidentata* (Niuxi),* Spatholobus stem* (Jixueteng),* Mulberry Twig* (Sangzhi),* Cassia Twig *(*Guizhi*),* Boswellia carteri *(Ruxiang), and* Commiphora myrrha Eng1* (Moyao) and animal medicines including hirudo nipponica Whitman (Shuizhi), Scorpio (Quanxie), and* Pheretima* (Dilong). NXT alleviates atherosclerosis involved in inhibition of iNOS expression and dendritic cell maturation [1, 2]. Clinical observation shows that NXT can increase antiplatelet effect and decrease subsequent major adverse cardiovascular events (MACE) in patients with cytochrome P450 2C19^*∗*^2 polymorphism undergoing percutaneous coronary intervention (PCI) [3]. Moreover, NXT reduces the development of diabetic retinopathy [4] and cardiomyocyte damage in response to reactive oxygen species (ROS) [5]; however, the mechanism of NXT on the cardiomyocyte apoptosis and autophagy in response to oxidative stress is still unclear.

Oxidative stress plays an important role in the pathogenesis of cardiovascular diseases. ROS is a main oxidative stress in cardiovascular damage. ROS is a byproduct of the normal metabolism of oxygen and has important roles in the cell signaling and homeostasis. But too much ROS production leads to oxidative stress [6]. The myocardial ischemia-reperfusion pathophysiologically produces ROS upon the restoration of blood flow [7]. ROS can be induced by hyperglycemia or H_2_O_2_ in vitro. At present, extracellular H_2_O_2_-induced H9c2 cell injury model is well documented [8]. Our previous study shows that the extract of* Angelica sinensis* and* Ligusticum chuanxiong* has protective effect against H_2_O_2_-induced endothelial cell damage [9] and inhibits rat vascular smooth muscle cell proliferation [10]. PPAR*α* plays a protective role in cardiomyocyte damage [11], while the mechanism of NXT water extract protecting cardiomyocyte from H_2_O_2_-induced damage is not well known. Here we found that NXT activated PPAR*α* signaling decreased H_2_O_2_-induced H9c2 cell apoptosis and autophagy.

## 2. Materials and Methods

### 2.1. NXT Water Extract

NXT was kindly provided by Xianyang Buchang Pharmaceutical Co. Ltd. (Shanxi, China). The NXT water extract was isolated from 5 g NXT powder incubating in 15 mL H_2_O 60°C for 6 hs, and then the solution was evaporated to 1 mL and filtered. The composition of NXT water extract was identified by UHPLC-Q-TOF Tandem Mass Spectrometry.

### 2.2. Cell Culture

The rat embryonic-heart derived H9c2 cell line (ATCC, CRL-1446). Cells were cultured at 37°C and 5% CO_2_ in 25 mL cell culture flask containing Dulbecco's Modified Eagle Medium (DMEM) (Gibco) supplemented with 10% (v/v) Fetal Bovine Serum (FBS) (Gibco), 100 U/mL penicillin, and 100 *μ*g/mL streptomycin sulfate. Cells were maintained in exponential phase of growth and were subcultured when they reached about 80% confluence at a split ratio of 1 : 3.

### 2.3. Cell Viability Assay

MTT assay was used to screen the cytotoxic or protective activity of the water extract compound from NXT. The cell viability was determined with MTT Cell Proliferation and Cytotoxicity Assay Kit (Sangon) according to the manufacturer's instructions. Cells (1 × 10^3^ cells/well) were seeded in 96-well plates. After the corresponding treatment, cells were washed twice with PBS and then incubated with the MTT solution for 1.5 h at 37°C. Cells were then dissolved in 200 *μ*L DMSO in 96-well plates. The absorbance of the reaction solution at 570 nm was measured with an ELISA-plate reader, Multidetection Microplate Reader Synergy H4 (BioTek).

### 2.4. Western Blot

Cells were seeded in 6-well plates and cultured as mentioned above. Cells were lysed in lysis buffer containing protease inhibitors. The lysate was sonicated (10 s) and then centrifuged at 13,000 ×g at 4°C for 10 min. Protein concentration in the supernatant was determined by the Pierce BCA Protein Assay Kit (Thermo). Samples were diluted (1 : 4) in protein loading buffer (67 mM Tris-HCl, pH 6.8, 30% glycerol, 2% SDS, and 0.01% bromophenol blue) and boiled for 5 min. The proteins were separated by SDS-PAGE using 10% acrylamide gels and transferred to a nitrocellulose membrane (PALL). After being blocked with 5% (w/v) nonfat dry milk dissolved in TBST buffer (10 mM Tris-HCl pH 7.5, 150 mM NaCl, and 0.1% Tween 20), the membranes were incubated with primary antibodies, anti-cascas3/9, anti-PARP-1, Bcl-2, Bax, Bad, and beta-actin (Santa Cruz Biotechnology) and LC3b antibody (Novus Biologicals). The membranes were washed three times with TBST buffer at room temperature and then incubated with HRP-conjugated secondary antibody for 1~2 h at room temperature. The secondary antibody was removed by washing three times with TBST buffer. The immunoreactive bands were visualized with ECL reagent.

### 2.5. Plasmids and Transfection

PPAR*α* shRNA plasmids (GV248 vector) were purchased from GeneCHEM (China). Plasmids were transfected by turboFect transfection reagent according to the manufacturer's instructions (Thermo Scientific).

### 2.6. Flow Cytometry (FCM) Assay

Cells were harvested by trypsinization and centrifuged at 400 ×g at 4°C for 10 min. For each sample, 10^6^ cells were collected. The cells were treated with Annexin V-FITC Apoptosis Detection Kit according to the manufacturer's instructions (BD Biosciences). The cells flow through the FCM at about 100–1000 cells per second.

### 2.7. Statistical Analysis

Data are expressed as the mean ± SEM. Statistical comparison was carried out with Student's *t*-test or one-way analysis of variance (ANOVA).

## 3. Results

### 3.1. Determination of Composition in NXT Water Extract by UHPLC-Q-TOF

The UHPLC-Q-TOF results show that the main composition of NXT water extract is paeoniflorin (80.4%), salvianolic acid B (10.1%), trihydroxybenzoic acid (4.6%), chlorogenic acid (1.8%), and ferulic acid (1.5%). The relative concentration of the main compositions was calculated (Table 1).

### 3.2. NXT Increases Cell Viability in Response to H_2_O_2_

**Figure 1:**
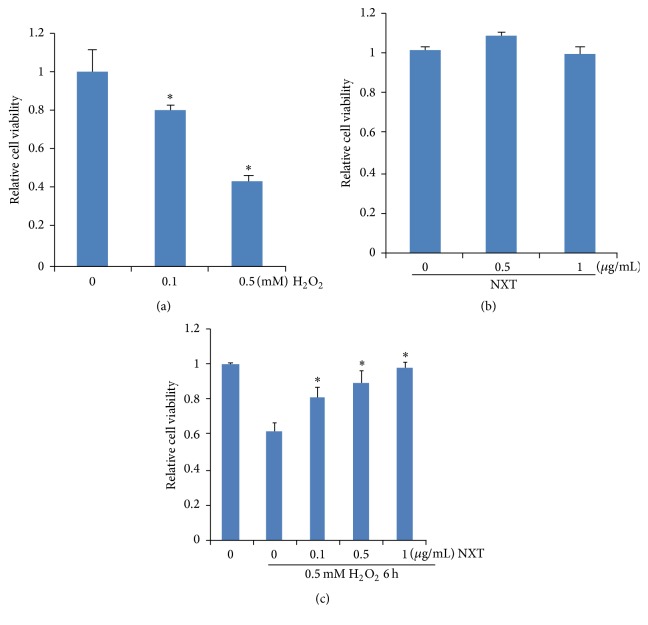
NXT extract increases cell viability. (a) H9c2 cells were treated with H_2_O_2_ as indicated for 6 h. Cell viability was assayed by MTT. Results are expressed as means ± SEM (*n* = 5). ^*∗*^ *P* < 0.05 versus no H_2_O_2_ treatment. (b) H9c2 cells were treated with NXT extract as indicated for 12 h. Cell viability was assayed by MTT. (c) H9c2 cells were pretreated with or without NXT extract for 12 h, and then cells were treated with 0.5 mM H_2_O_2_ for 6 h as indicated. Cell viability was assayed by MTT. Results are expressed as means ± SEM (*n* = 5). ^*∗*^ *P* < 0.05 versus no NXT treatment.

H9c2 cells were treated with H_2_O_2_. The results show that H_2_O_2_ dose dependently reduced cell viability (Figure 1(a)). Further analysis shows that the NXT extract had no cytotoxicity effect on H9c2 cells (Figure 1(b)). To assay the protective effect of NXT on H9c2 cells, cells were pretreated with different dose (0.1~1.0 *μ*g/mL) of NXT for 12 h. After that cells were induced with H_2_O_2_ for 6 h. The results show that NXT significantly increased cell survival in response to H_2_O_2_ (Figure 1(c)).

### 3.3. NXT Increases Antiapoptotic Protein Expression in Response to H_2_O_2_

**Figure 2:**
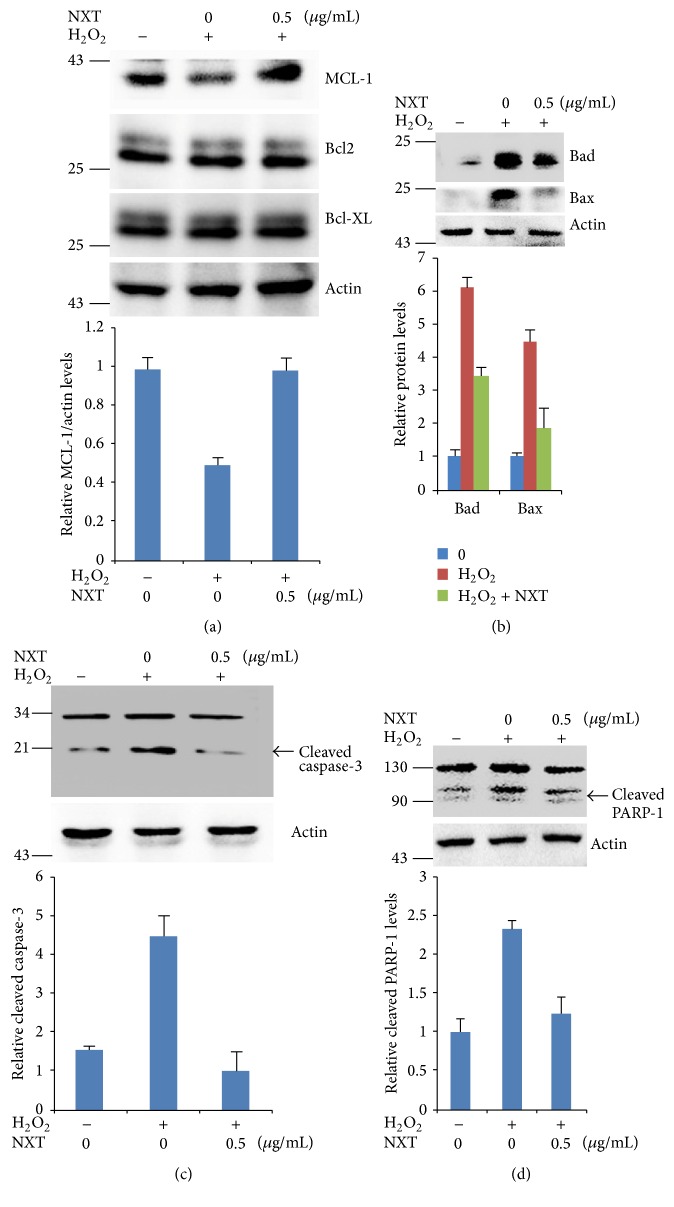
NXT increases antiapoptotic protein levels. (a) H9c2 cells were pretreated with or without 0.5 *μ*g/mL NXT for 12 h, and then the cells were treated with 200 *μ*M H_2_O_2_ for 6 h. Cell lysates were subjected to Western blot. (b) H9c2 cells were pretreated with or without 0.5 *μ*g/mL NXT for 12 h, and then the cells were treated with 200 *μ*M H_2_O_2_ for 6 h. Cell lysates were subjected to Western blot as indicated. (c) H9c2 cells were pretreated with or without 0.5 *μ*g/mL NXT for 12 h, and then the cells were treated with 200 *μ*M H_2_O_2_ for 6 h. Cell lysates were subjected to Western blot as indicated. (d) H9c2 cells were pretreated with or without 0.5 *μ*g/mL NXT for 12 h, and then the cells were treated with 200 *μ*M H_2_O_2_ for 6 h. Cell lysates were subjected to Western blot. Data are triplicates from three independent experiments.

The antiapoptotic MCL-1 protein prevents the release of mitochondrial contents against caspase-3 activation [12, 13]. We next detected the effect of NXT on the cell apoptotic signaling. The results show that NXT extract significantly increased antiapoptotic MCL-1 protein levels (Figure 2(a)). In contrast, NXT decreased Bad and Bax proapoptotic protein levels (Figure 2(b)). H_2_O_2_ induces cell apoptosis through activate caspase-3 [12, 13]. Our results show that NXT extract reversed this apoptotic signaling by inhibiting caspase-3 activation (Figure 2(c)); subsequently, cleaved PARP-1 was decreased in H9c2 cells treated with NXT extract (Figure 2(d)). These findings suggest that NXT inhibited the H9c2 cell apoptosis through inhibiting activation of caspase-3/PARP-1 signaling pathway.

### 3.4. NXT Decreases Cell Apoptosis

**Figure 3:**
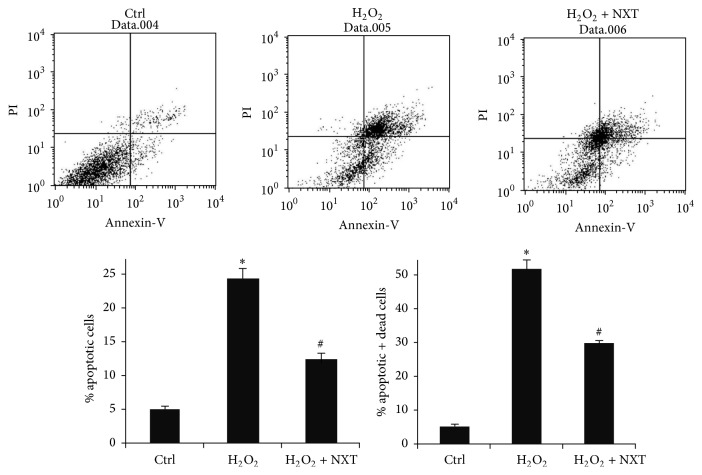
NXT extract inhibits H_2_O_2_-induced cell apoptosis. H9c2 cells were pretreated with or without 0.5 *μ*g/mL NXT for 12 h, and then the cells were treated with 200 *μ*M H_2_O_2_ for 6 h. Cell apoptosis was assayed by FCM. Apoptotic cells or apoptotic and death cells were quantified. ^*∗*^ *P* < 0.05 Ctrl versus H_2_O_2_ only treatment; ^#^ *P* < 0.05 H_2_O_2_ versus NXT treatment. Data are triplicates from three independent experiments.

To detect NXT protective effect on H9c2 cells in response to H_2_O_2_, flow cytometry analysis was performed to detect the effect of NXT on H_2_O_2_-induced H9c2 cell apoptosis. The results show that H_2_O_2_ significantly induced cell apoptosis, but NXT reversed this event (Figure 3), suggesting that NXT extract inhibited H_2_O_2_-induced H9c2 cell apoptosis associated with increased antiapoptotic protein levels.

### 3.5. NXT Inhibits Cell Apoptosis by Increasing PPAR*α* Expression

**Figure 4:**
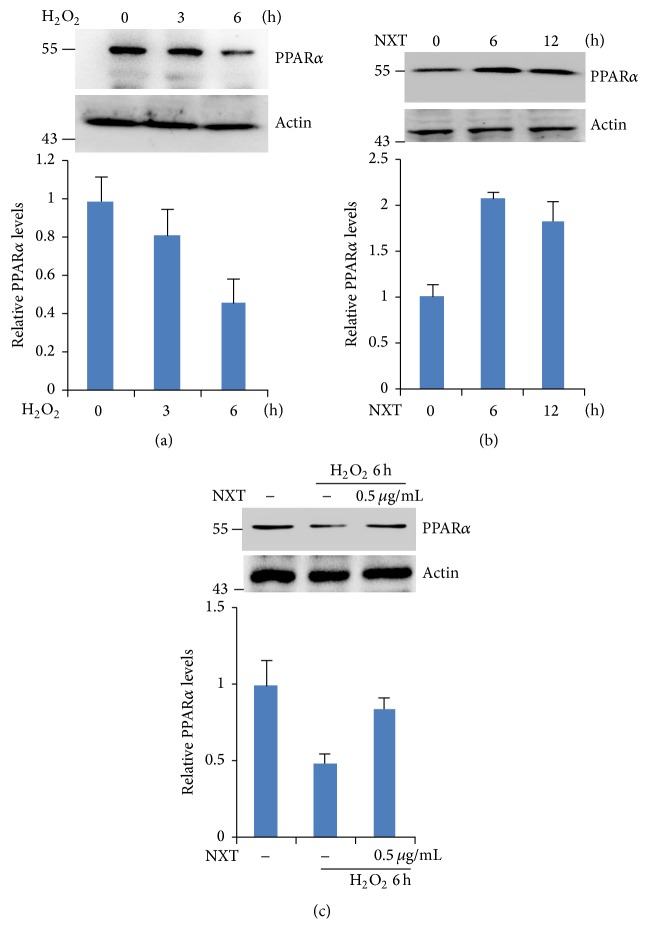
NXT extract increases PPAR*α* expression. (a) H9c2 cells were treated with 200 *μ*M H_2_O_2_ as indicated time. Cell lysates were subjected to Western blot. (b) H9c2 cells were treated with 0.5 *μ*g/mL NXT as indicated time. Cell lysates were subjected to Western blot. (c) H9c2 cells were treated with or without 0.5 *μ*g/mL NXT for 12 h. After that, cells were treated with 200 *μ*M H_2_O_2_ for 6 h. Cell lysates were subjected to Western blot. Data are triplicates from three independent experiments.

**Figure 5:**
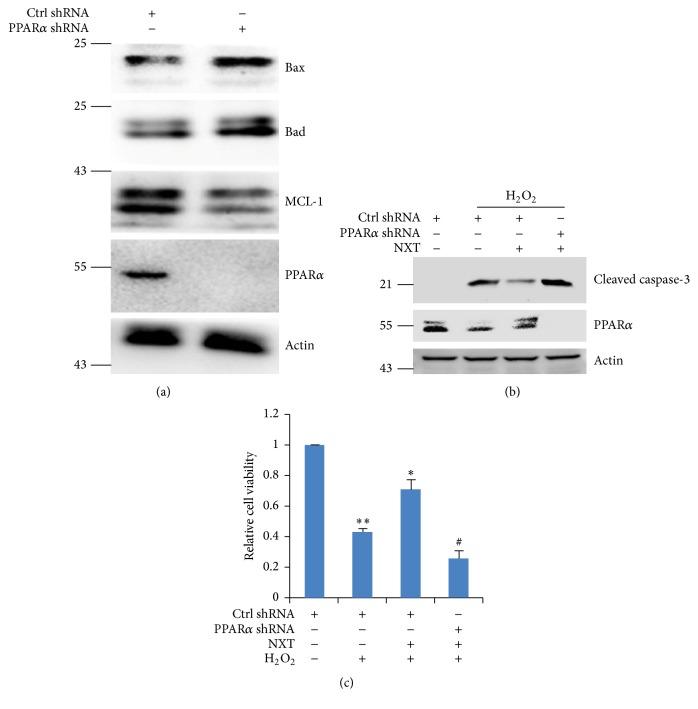
Silenced PPAR*α* decreases NXT protective effect on H9c2 cells. (a) H9c2 cells were transfected with control shRNA or PPAR*α* shRNA for 24 h. Cell lysates were subjected to Western blot. Data are triplicates from three independent experiments. (b) H9c2 cells were transfected with control shRNA or PPAR*α* shRNA for 24 h. Cells were pretreated with or without 0.5 *μ*g/mL NXT for 12 h, and then cells were treated with 200 *μ*M H_2_O_2_ for 6 h. Cell lysates were subjected to Western blot. Data are triplicates from three independent experiments. (c) H9c2 cells were transfected with control shRNA or PPAR*α* shRNA for 24 h. Cells were pretreated with or without 0.5 *μ*g/mL NXT for 12 h, and then cells were treated with 200 *μ*M H_2_O_2_ for 6 h. Cell viability was assayed by MTT. Results are expressed as means ± SEM (*n* = 5). ^*∗∗*^ *P* < 0.05 versus control (no treatment); ^*∗*^ *P* < 0.05 versus only H_2_O_2_ treatment. ^#^ *P* < 0.05 versus only H_2_O_2_ treatment.

Our data have demonstrated that NXT significantly decreased H9c2 cell apoptosis in response to H_2_O_2_, which was involved in increasing antiapoptotic protein expression. As nuclear receptor, PPAR is a critical regulator of inflammation, adipocyte differentiation, and glucose homeostasis [3, 14–17]. Other reports show that PPAR*α* protects against cardiomyocyte damage [11, 18]. Here we found that H_2_O_2_ significantly reduced PPAR*α* protein levels (Figure 4(a)), but NXT increased PPAR*α* protein expression levels (Figure 4(b)). More importantly, cells pretreated with NXT did not reduce PPAR*α* protein levels in response to H_2_O_2_ (Figure 4(c)). Further analysis shows that PPAR*α* silenced H9c2 cells increased proapoptotic protein levels (Bax and Bad) and decreased antiapoptotic protein MCL-1 protein levels (Figure 5(a)). Consistent with this, PPAR*α* silenced H9c2 cells led to increased activation of caspase-3 in response to H_2_O_2_ (Figure 5(b)). As expected, PPAR*α* silence in H9c2 cells did not increase cell survival in response to H_2_O_2_ (Figure 5(c)). These findings show that NXT activated PPAR*α* singling leading to inhibition of H_2_O_2_-induced H9c2 cell damage.

### 3.6. NXT Reduces Cell Autophagy in Response to H_2_O_2_

**Figure 6:**
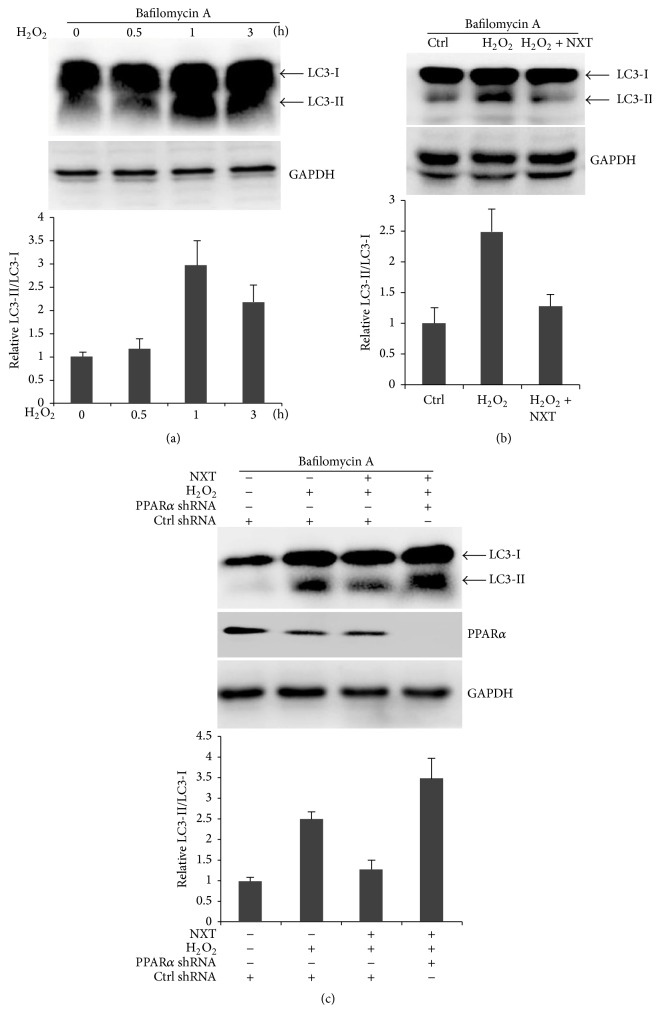
NXT/PPAR*α* signaling inhibits cell autophagy. (a) H9c2 cells were treated with 200 *μ*M H_2_O_2_ as indicated time. Cell lysates were subjected to Western blot. (b) H9c2 cells were treated with or without 0.5 *μ*g/mL NXT for 12 h. After that, cells were treated with 200 *μ*M H_2_O_2_ for 6 h. Cell lysates were subjected to Western blot. (c) H9c2 cells were transfected with control shRNA or PPAR*α* shRNA for 24 h. Cells were pretreated with or without 0.5 *μ*g/mL NXT for 12 h, and then cells were treated with 200 *μ*M H_2_O_2_ for 6 h. Cell lysates were subjected to Western blot. Cells were treated with 50 *μ*M bafilomycin A for 1 h before cell lysis. Data are triplicates from three independent experiments.

Mammalian cell death has three types: apoptosis, necrosis, and autophagy [12]. We further detected whether H_2_O_2_ would induce cardiomyocyte autophagy. As expected, H_2_O_2_ significantly increased processing of LC3-I to LC3-II conversion (Figure 6(a)), suggesting that H_2_O_2_ induced H9c2 cell autophagy. Further analysis shows that H_2_O_2_-induced cell autophagy was reduced by NXT treatment (Figure 6(b)), suggesting that NXT decreased H_2_O_2_-induced H9c2 cell autophagy. As PPAR*α* inhibited H_2_O_2_-induced cell apoptosis, further analysis shows that PPAR*α* silence led to increased H9c2 cell autophagy (Figure 6(c)), suggesting that PPAR*α* suppressed cell autophagy in response to H_2_O_2_.

## 4. Discussions

Naoxintong (NXT) is an empirical formula based on the principle of traditional Chinese medicine, which has been approved by China Food and Drug Administration (CFDA) and is widely used for treatment of patients with cerebrovascular and cardiovascular diseases in China. Increasing evidences show that NXT alleviates atherosclerosis involved in reducing expression of iNOS and NO level in the vessel wall [2]. In the mice model of atherosclerosis, NXT suppresses atherosclerosis through lipid-lowering and inhibition of dendritic cell maturation [1]. Clinical observations show that NXT can increase the antiplatelet effect and decrease subsequent major adverse cardiovascular events (MACE) in patients with cytochrome P450 2C19^*∗*^2 polymorphism undergoing percutaneous coronary intervention (PCI) [3]. In addition to that, NXT reduces the development of diabetic retinopathy involved in inhibition of the expression of CAS-3, MMP-2/9, and TNF-*α* [4]. Moreover, NXT increases the effect on the catalytic activities of drug-metabolising CYP2C19 enzyme [19]. Although ethanol extraction of NXT reduces cardiomyocyte cell damage [5], the water soluble components of NXT on H_2_O_2_-induced cardiomyocyte cell apoptosis are still unclear. Here we found that the NXT water extract has significantly protective effect on H9c2 cells in response to H_2_O_2_ treatment. To detect the possible component of the NXT water extract, the UHPLC-Q-TOF analysis was performed. Interestingly, the main composition of NXT water extract is paeoniflorin (80.4%), salvianolic acid B (10.1%), trihydroxybenzoic acid (4.6%), chlorogenic acid (1.8%), and ferulic acid (1.5%) (Table 1). These findings suggest that paeoniflorin may be the main or critical effective component for cardiovascular diseases, which needs to be further determined.

MCL-1 is the antiapoptotic proteins, but Bad and Bax are proapoptotic proteins, and MCL-1 inhibits caspase-3-mediated cell apoptotic signaling pathway [20, 21]. It is well known that activated caspase-3 results in cleavage of PARP-1 leading to cell apoptosis [22]. Some natural compounds from traditional medicine increase Bcl-2 family protein levels to inhibit cell apoptosis [23]. Our data show that NXT increased MCL-1 protein levels and decreased activation of caspase-3 and PARP-1 in H_2_O_2_-treated H9c2 cells. These results show that NXT protected the cells against apoptosis by reducing caspase-3/PARP-1 signaling pathway. The increased antiapoptotic Bcl-2 family proteins (MCL-1) and decreased proapoptotic proteins (Bad and Bax) suggest that NXT extract reduced cell apoptosis in response to H_2_O_2_. FCM assay further demonstrated that NXT extract decreased H9c2 cell apoptosis. MCL-1 is transmembrane protein in the mitochondria; however, Bax and Bak are two nuclear-encoded proteins that are able to pierce the mitochondrial outer membrane to induce apoptosis [24]. Based on our findings, mitochondria may be one of the targets of NXT water extract.

As nuclear receptor, PPARs are the critical regulator of inflammation, adipocyte differentiation, and glucose homeostasis [3, 14–17]. Other reports show that PPAR*α* protects against cardiomyocyte damage [11, 18]. Here we found that NXT significantly increased PPAR*α* expression levels in response to H_2_O_2_. In contrast, silenced PPAR*α* terminated the inhibition of NXT on H_2_O_2_-induced H9c2 cell damage involved in PPAR*α* mediated the MCL-1 antiapoptotic protein expression.

Autophagy delivers cytoplasmic materials or organelles into lysosomes for degradation, which is also a progress of nutrient recycling [25]. Especially in response to starvation stress, autophagy increases cell survival; however, long-term periods autophagy without new nutrients replenishment leads to digestion of all available substrates and death (autophagy-associated cell death) [12, 25]. Here we found that H_2_O_2_ significantly induced H9c2 cell autophagy. In contrast, NXT reversed this event. More importantly, NXT reduced H9c2 cell autophagy in a PPAR*α* dependent manner, suggesting that NXT/PPAR*α* signaling suppressed H9c2 cell autophagy.

## 5. Conclusion

NXT water extract inhibited H9c2 cell apoptosis and autophagy by increasing PPAR*α* expression leading to increased H9c2 cell viability.

## Figures and Tables

**Table 1:** Diverse composition in NXT water extract identified by UHPLC-Q-TOF.

Number	Peak number	Identification	Formula	MS	Relative concentration (%)
1	35	Paeoniflorin	C_23_H_28_O_11_	480.1632	80.42
2	55	Salvianolic acid B	C_36_H_30_O_16_	718.1534	10.13
3	11	Gallic acid	C_7_H_6_O_5_	170.0215	4.59
4	22	Chlorogenic acid	C_16_H_18_O_9_	354.0951	1.79
5	39	Ferulic Acid	C_10_H_10_O_4_	194.0579	1.53
6	25	Hydroxysafflor yellow A	C_27_H_32_O_16_	612.1690	0.80
7	49	Rosmarinic acid	C_18_H_16_O_8_	360.0845	0.22
8	16	5-Hydroxymethylfurfural	C_6_H_6_O_3_	126.0317	0.17
9	47	1,5-Di-O-caffeoylquinic acids	C_25_H_24_O_12_	516.1268	0.14
10	46	3,5-Di-O-caffeoylquinic acids	C_25_H_24_O_12_	516.1268	0.11
11	48	Kaempferol-3-O-rutinoside	C_27_H_30_O_15_	594.1585	0.07
12	51	Lithospermate	C_27_H_22_O_12_	538.1111	0.03
